# The Biomechanical Analysis of Magnitude and Direction of Force by Different Techniques of Thoracic Spinal Manipulation

**DOI:** 10.1155/2020/8928071

**Published:** 2020-07-26

**Authors:** Sunghee Joo, Junghyun Kim, Yongwoo Lee, Changho Song

**Affiliations:** Department of Physical Therapy, College of Health Science, Sahmyook University, Republic of Korea

## Abstract

**Background:**

Spinal manipulation (SM) has been widely recognized and used with success in health care fields for spinal joint dysfunction and pain. SM is a procedure that involves small amplitude manipulative thrusts performed with speed. These forces are complex three-dimensional (3-D) forces delivered to create forces and moments at the joint of interest to cause joint movements. The aim of this study was to conduct a 3-dimensional analysis of the magnitude and direction of the forces transmitted in 2 techniques of thoracic spinal manipulation (TSM). *Materials/Methods*. Thirty-two healthy participants were recruited from the university community. The physical therapist performed TSM using anterior (A) to posterior (P) and P to A techniques once at each of T3, T7, and T12 spinal levels. The magnitude and direction of the forces transmitted during TSM were sensed by the force plates, and the camera system monitored vertebral motion by tracking motion markers.

**Results:**

There were no significant differences on the *x*-axis while there were significant differences on the *y*-axis between the measured spinal levels in the P to A technique. There were significant differences found at preload force maximum, preload force minimum, and peak force between T3 and T12 and between T7 and T12 and at peak base force between T7 and T12 on the *z*-axis. In the A to P technique, there were significant differences in the change of force in measured spinal levels at different axes.

**Conclusion:**

These study findings can help therapists better understand the mechanism of TSM and enhance the clinical usefulness of TSM.

## 1. Background

Spinal manipulation (SM) has been widely recognized and used with success in health care fields over the past century as a conservative treatment modality for spinal joint dysfunction and pain [1, 2]. SM is a procedure that involves small amplitude manipulative thrusts performed with speed [3]. It is commonly performed by physical therapists, osteopaths, chiropractors, and medical practitioners [4]. The clinician performs timely weight transfer, with partial support for his/her body weight, from the lower extremities to the upper extremities, and the force is transmitted to the patient [5]. These forces are complex three-dimensional (3-D) forces delivered to create forces and moments at the joint of interest to cause joint movements. This type of manipulation is known as High-Velocity Low-Amplitude SM (HVLA-SM) [6, 7].

SM techniques have long been studied for their clinical effectiveness. Most randomized controlled clinical trials have been conducted using HVLA thrusts in parents with low back and neck pain and headache [8–10]. Although clinical outcome studies have increased attention in HVLA-SM, basic evidence of biomechanical mechanisms to support the clinical effectiveness is lacking [11]. It is important that physical therapists understand the biomechanical differences and quantification of HVLA-SM to enable them to practice SM safely and decrease adverse patient outcomes. Therefore, quantitative investigations into the dynamics of SM have been conducted for centuries. Most of the studies only assessed the normal force (defined by the perpendicular force to the back surface) and the characteristics of the thrusting hand in the vertical axis but ignored the various body planes with regard to weight bearing and the complex loading of the spine [11–14].

The safety of TSM has been an issue of significant debate over the past decade. Puentedura et al. [15] and Carnes et al. [16] reported the side effects of TSM which include pain, headache, discomfort, and fatigue. Stemper et al. [17] investigated some concerns about the strength and depth of TSM. Findings from the above-mentioned studies led to our reconsideration of the relative safety of TSM. Furthermore, there were studies that quantified the biomechanical effects of SM on the thoracic spine and compared them to those on the cervical and lumbar spines. This was probably due to the technical difficulties met in the movement analysis of the thoracic spine [18]. Forand et al. [12] studied the forces generated by manipulating a transverse process in the vicinity of T4 and T9 using Emed pressure sensors, and Triano et al. [5] did pioneering work on 3-D load transmission using an inverse dynamics approach with a force plate embedded in the treatment table during SM. These studies reported the force applied on the preload force, peak force, thrust duration, and rates of loading from the force-time data collected during SM. However, they did not report the direction of force.

It is suggested that further studies be conducted to understand the operation and interaction of these properties during joint manipulation in multidimensional axes [19, 20]. Therefore, the purpose of this study was to quantify the magnitude and direction of the forces transmitted in 2 TSM techniques in 3-D motion.

## 2. Materials and Methods

### 2.1. Subjects

Thirty-two healthy participants were recruited via board postings in the Sahmyook university community. The inclusion criteria were as follows: (1) absence of pain or weakness in the spine, (2) absence of musculoskeletal symptoms in the last 6 months, and (3) absence of open wounds on the back. Participants were excluded if they had any of the following: (1) a history of thoracic spine surgery, (2) signs of central nervous system involvement, and (3) contraindications to manipulation, such as osteoporosis, metastatic disease, or systemic arthritis [21]. All participants completed an intake questionnaire containing questions on health screening, demographics, and symptom history. They were also provided verbal and written explanations of the study procedures, and they signed the informed consent form prior to participation. The University of Sahmyook Human Research Ethics Committee approved this study (2-7001793-AB-N-012018131HR).

### 2.2. Procedure

Thirty-two healthy participants were recruited for the study. This study was a cross-sectional study designed to measure the magnitude and direction of the forces transmitted during TSM, which was conducted in the motion analysis laboratory of Sahmyook University. Two force plates were positioned beneath the treatment table to measure the magnitude and direction of forces transmitted during TSM. The length of the 2 force plates spanned from around the head of the participant to around his/her knee. For posterior to anterior (P to A) TSM, the participants were instructed to lie in prone position on the instrumented treatment table with face and arms at rest, but for anterior to posterior (A to P) TSM, they were instructed to lie in supine position with arms crossed. The spinous processes of T3, T7, and T12 were the standardized motion marker locations used for P to A TSM. The scapular inferior angle and the root of the scapular spine were used as skeletal landmarks. The markers were attached to the spot using double-sided tape. A single manual therapist with clinical experience placed the markers to maximize palpatory accuracy and then performed TSM. The magnitude and direction of the forces transmitted during TSM were sensed by the force plate. The camera system monitored the vertebral motion by tracking the motion markers.

### 2.3. TSM: A to P

**Figure 1:**
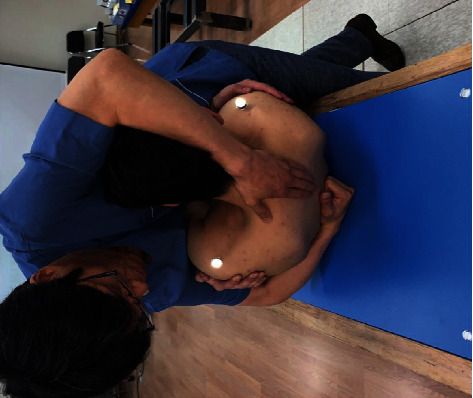
Anterior to posterior thoracic spinal manipulation (TSM).

For this study, 2 TSM techniques (A to P and P to A) were performed once at each of T3, T7, and T12. A single TSM on each spinal level was used to standardize the intervention. To perform A to P TSM, the participants were instructed to adjust their thoracic spine and lie supine on the force plate placed beneath the instrumented treatment table. They were then instructed to cross their arms with each hand placed on the opposite shoulder so that 1 elbow is on top of the other in front of the lower chest. The physical therapist was instructed to perform supine TSM using a clenched fist for posterior contact. The participant's position was stabilized, and he/she was asked to inhale and fully exhale. At the end of the respiratory cycle, the adjustive impulse of supine TSM was generated by thrusting with the weight of the therapist's torso through the patient and toward the posterior contact. The supine TSM accelerated the participant toward the posterior contact and generated a force back toward the participant's spine as the posterior contact meets the firm resistance of the instrumented treatment table (Figure 1).

### 2.4. TSM: P to A

**Figure 2:**
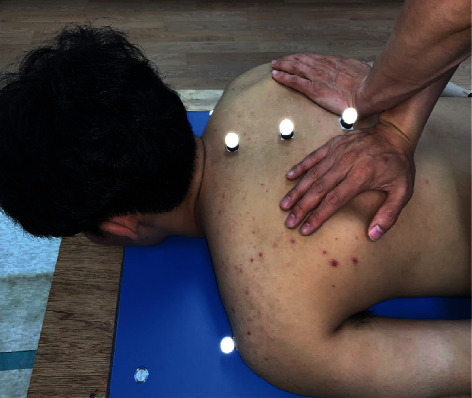
Posterior to anterior thoracic spinal manipulation (TSM).

To perform P to A TSM, participants were instructed to adjust their thoracic spine and lie in a prone position with the face and arms at rest and the force plate placed beneath the instrumented treatment table. The physical therapist was instructed to stand on the left side of the participant and perform prone TSM using the bilateral knife contact technique which is considered common in clinical practice. The therapist crossed his forearms and made contact with both transverse processes of the target spinal level using the pisiform bones to ensure proper contact. The participant's position was stabilized, and he/she was asked to inhale and fully exhale. At the end of the respiratory cycle, the therapist delivered a posterior to anterior impulse to the target spinal level (Figure 2).

### 2.5. Measurements

**Figure 3:**
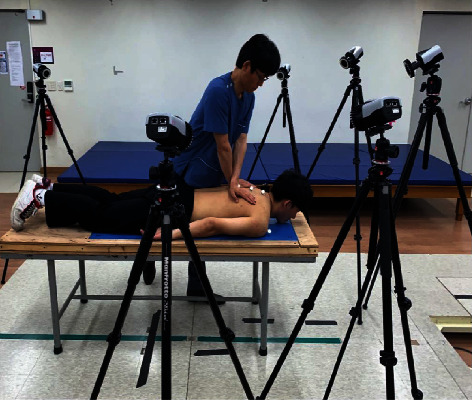
Infrared cameras.

The force plate variables of interest were the magnitude and direction of the forces during TSM. Two force plates (Type 9266AA, Kistler Instrument AG, Switzerland, 2008) positioned beneath the treatment table were used to record the reaction loads transmitted while performing TSM. One was placed on the upper part of participant's body, and the other was placed on the lower part. The force plates can sense forces and moments about the *x*-, *y*-, and *z*-axes [22]. They recorded the reaction loads passing through a participant's body including the sum of applied loads from the manual therapist. Signals from the force plates were amplified by 4000 and passed to a commercial software system (TestPoint, Capital Equipment Corp., Billerica, Mass) for calculation of loads, display, and storage. Six infrared cameras (Qqus 1 series, Qualisys AB, Sweden, 2012) were used for monitoring vertebral motion by tracking 3 markers in P to A TSM. In A to P TSM, the markers could not be attached to the vertebral segments due to the close physical contact between the therapist and the subject. We attached markers on acromions of both shoulders, and the markers could indicate the direction of the head (Figure 1). The kinematic parameters stored by the camera were analyzed using Qualisys Track Manager (Track Manager version 2.5, Qualisys, Sweden, 2012) (Figure 3).

### 2.6. Statistical Analysis

**Figure 4:**
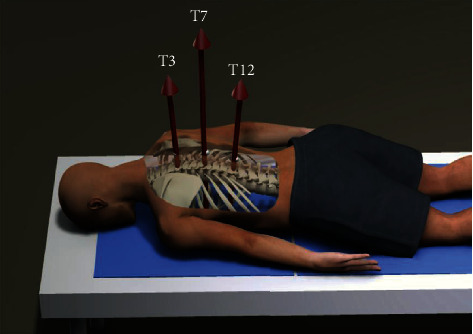
Comparison of force according to the spine level.

TSM force data from the force plates and camera were collected using PowerLab data acquisition and Chart software version 4.2.4 (ADInstruments, Castle Hill, Australia) and calibrated using Qualisys Track Manager (Track Manager version 2.5, Qualisys). In this study, *x*-, *y*-, and *z*-axes were the lateral, horizontal, and vertical coordinates, respectively, for the determination of the magnitude and direction of forces transmitted during TSM in 3 dimensions [21]. The *x*-axis represents the criteria for the left and right sides of participants lying in prone position, and the force applied to the right side is expressed as a positive number. The *y*-axis represents the reference to the cephalad and caudal directions, and the force applied caudally is expressed as a positive number. The *z*-axis represents the vertical direction, and the force exerted on the abdominal area of the participant is expressed as a positive number. As Campbell and Snodgrass [23] suggested, manipulation force-time history graphs were used to determine data points for TSM analysis. Five biomechanical parameters were identified: preload force maximum (N), preload force minimum (N), peak force (N), thrust duration (seconds), and thrust rate (N/second). Statistical analysis of all collected data was performed using the SPSS software (version 20.0 for Windows; SPSS Inc., Chicago, IL, USA), with the significance level set at a *p* value < 0.05. A two-way repeated measures analysis of variance (ANOVA) was used to compare force on 3-D axes for each spinal level. The post hoc Tamhane analysis was used for multiple comparisons (Figure 4).

## 3. Results

A total of 32 health subjects (20 males, 12 females) took part in this study. The general characteristics of the subjects were as follows: 24.94 ± 6.05 years, 168.40 ± 9.52 cm, and 65.05 ± 14.03 kg. There were no significant differences observed on the *x*-axis between the measured spinal levels in the P to A technique (Table 1). Significant differences were observed between all measured spinal levels on the *y*-axis (Table 2). There were significant differences found at preload force maximum, preload force minimum, and peak force between T3 and T12 and between T7 and T12 and at peak base force between T7 and T12 on the *z*-axis (Table 3). In the A to P technique, there were significant differences in the change of force in measured spinal levels at baseline and peak-baseline forces on the *x*-axis (Table 4). Significant differences were observed at the peak force level between T3 and T12 and in all parameters of force between T3 and T7 on the *y*-axis (Table 5). There were significant differences at baseline and peak forces between T3 and T12 and in all parameters of force between T3 and T12 and between T7 and T12. Significant differences were also found at baseline and peak-baseline forces between T3 and T7 (Table 6).

## 4. Discussion

This study investigated the changes of biomechanical characteristics in 2 TSM techniques. Our results showed that there were statistically significant differences in the magnitude and direction of forces between spinal levels and techniques on 3-D kinematics.

In quantifying the forces transmitted during the P to A technique, no significant differences were noted in the magnitudes of the left and right forces between different spinal levels on the *x*-axis. This means that the force was applied equally to the left and the right. It is worth noting that, based on the data presented in Table 3, the force at T3 (40.35 ± 16.71 N) was directed caudally but the force at T12 (−35.56 ± 12.54 N) in the cephalad direction, and the force directed caudally is expressed as a positive number on the *y*-axis. The maximum forces in the P to A technique showed at T7 to be 487.90 ± 71.98 N, next at T3 to be 458.71 ± 92.64 N, and then at T12 to be 432.30 ± 47.042 N on the *z*-axis. These results of maximum force are similar to those of earlier studies on thoracic manipulation which reported peak forces between 200 and 800 N [12, 24].

Downie et al. [25] reported that most TSM kinetic studies used the P to A technique because 3-D quantifications of TSM kinetics are more complex with any other technique. It was difficult to quantify the magnitude and direction of the forces in our study using the A to P technique because the standard deviation was large. There were no preload force maximum or preload force minimum parameters for the A to P technique as shown in Tables 4, 5, and 6. The preload force maximum and preload force minimum values were generated by contact in the P to A technique; however, there were no such processes in the A to P technique as it was performed with the subject in a maintained position of segmental flexion with posterior contact [26]. Furthermore, the data values obtained are rarely used because of the presence of large deviations. It was therefore meaningless to distinguish the preload force maximum and preload force minimum in the A to P technique. We calculated the angular kinematics during HVLA-SM of the thoracic spine. It was calculated by a tilted angle using the value of *z*, which is the vertical direction, and the value of *y*, which is the cephalad to caudal direction. It was -5.03° at T3, -1.58° at T7, and 4.71° at T12 in the P to A technique. However, the values of angular kinematics in the A to P technique were <1° at all measured spinal levels. These results suggest that, in the A to P technique, the direction of the force tends to be vertical even if the therapist adjusts the angle of the subject's spine. This hypothesis is supported by the results shown in Tables 2 and 5. The spinal levels showed fixed directions, and significant differences were observed at all measured spine levels in the P to A technique. In contrast, the values in the A to P technique did not show fixed directions on the *y*-axis. As shown in Table 6, the magnitudes of the forces recorded were considerably larger in the A to P technique at T3 (735.57 ± 148.24 N), at T7 (769.45 ± 140.50 N), and at T12 (719.33 ± 131.73 N) spinal levels compared to the values at the same levels in the P to A TSM technique (458.71 ± 92.64 N at T3, 487.90 ± 71.98 N at T7, and 432.30 ± 47.04 N at T12). These results could be obtained by two possible reasons. Firstly, the A to P technique entails force generation through the manipulation of the weights of the subject and the therapist. Secondly, the force transition in P to A TSM will be partially offset by the inertial forces of the upper torso masses before being captured by the sensors of the force plate. These findings suggest that the A to P technique is more effective than the P to A technique if the magnitude of force alone is considered.

The clinical significance of the biomechanical differences highlighted in this study is related to the different facet orientations at consecutive intervertebral levels and the geometric strains on anatomical thoracic vertebral structures [20, 27, 28]. As far as we know, this study was the first to quantify TSM angular kinematics and the magnitude and direction of forces transmitted in the A to P technique. Our biomechanical analysis provided a rational basis for standardizing or identifying parameters that should be used to distinguish between the TSM techniques used in clinical trials. However, our study results were not standardized because the TSM was performed by a single therapist. Therefore, future research should investigate the biomechanical differences of forces transmitted during HVLA-TSM procedures performed by different therapists.

## 5. Conclusion

This study provided the biomechanical 3-D analysis of forces in 2 TSM techniques. The results showed that there were significant differences in the change of force-time at measured spinal levels during TSM and between spinal levels in 3-D axes. Our study findings will help therapists better understand the mechanism of TSM and enhance the clinical usefulness of TSM.

## Figures and Tables

**Table 1:** Comparison of force according to spine level on the *x*-axis in P to A TSM (*N* = 32).

	T3	T7	T12	*F*
Baseline	2.28 ± 7.38	3.61 ± 7.61	1.87 ± 6.33	2.478
PreMax	13.17 ± 16.11	9.57 ± 11.84	8.82 ± 7.85	1.868
PreMin	10.28 ± 14.60	6.81 ± 10.26	5.47 ± 7.16	1.930
Peak	13.32 ± 17.74	16.46 ± 16.02	11.48 ± 11.08	3.121
Peak-baseline	11.04 ± 15.05	12.85 ± 13.68	9.61 ± 9.16	1.193

Note. a = significant difference between T3 and T7; b = significant difference between T7 and T12; c = significant difference between T3 and T12; PreMax = preload maximum; PreMin = preload minimum; T = thoracic spine level. Values are expressed as mean ± standard deviation (N).

**Table 2:** Comparison of force according to the spine level on the *y*-axis in P to A TSM (*N* = 32).

	T3	T7	T12	*F*	Post hoc
Baseline	25.39 ± 11.45	19.29 ± 13.70	19.13 ± 12.75	22.338	
PreMax	49.29 ± 20.50	25.18 ± 18.28	−5.11 ± 13.84	220.415^∗^	a, b, c
PreMin	45.84 ± 18.62	28.14 ± 17.60	2.56 ± 14.37	138.381^∗^	a, b, c
Peak	65.74 ± 23.69	32.70 ± 21.60	−16.43 ± 17.84	343.997^∗^	a, b, c
Peak-baseline	40.35 ± 16.71	13.42 ± 13.65	−35.56 ± 12.54	337.236^∗^	a, b, c

Note. a = significant difference between T3 and T7; b = significant difference between T7 and T12; c = significant difference between T3 and T12; PreMax = preload maximum; PreMin = preload minimum; T = thoracic spine level. Values are expressed as mean ± standard deviation (N).

**Table 3:** Comparison of force according to the spine level on the *z*-axis in P to A TSM (*N* = 32).

	T3	T7	T12	*F*	Post hoc
Baseline	377.81 ± 81.38	371.06 ± 78.10	308.20 ± 67.27	301.297^∗^	a, b, c
PreMax	653.41 ± 122.81	670.75 ± 125.19	568.76 ± 90.60	22.884^∗^	b, c
PreMin	591.50 ± 72.78	599.65 ± 79.16	532.20 ± 93.01	22.576^∗^	b, c
Peak	836.53 ± 122.95	858.96 ± 123.19	740.50 ± 102.12	80.971^∗^	b, c
Peak-baseline	458.71 ± 92.64	487.90 ± 71.98	432.30 ± 47.04	14.047^∗^	b

Note. a = significant difference between T3 and T7; b = significant difference between T7 and T12; c = significant difference between T3 and T12; PreMax = preload maximum; PreMin = preload minimum; T = thoracic spine level. Values are expressed as mean ± standard deviation (N).

**Table 4:** Comparison of force according to the spine level on the *x*-axis in A to P TSM (*N* = 32).

	T3	T7	T12	*F*	Post hoc
Baseline	−7.79 ± 8.47	−1.55 ± 9.84	5.08 ± 13.13	15.933^∗^	a, b, c
Peak	74.34 ± 22.52	69.33 ± 26.73	62.21 ± 24.83	5.005^∗^	c
Peak-baseline	82.13 ± 25.24	70.88 ± 29.58	57.13 ± 22.70	20.482^∗^	a, b, c

Note. a = significant difference between T3 and T7; b = significant difference between T7 and T12; c = significant difference between T3 and T12; PreMax = preload maximum; PreMin = preload minimum; T = thoracic spine level. Values are expressed as mean ± standard deviation (N).

**Table 5:** Comparison of force according to the spine level on the *y*-axis in A to P TSM (*N* = 32).

	T3	T7	T12	*F*	Post hoc
Baseline	11.44 ± 10.02	6.64 ± 9.30	−2.51 ± 7.21	20.482^∗^	a, b, c
Peak	9.92 ± 20.95	−8.09 ± 17.92	−11.47 ± 22.21	10.095^∗^	a, c
Peak-baseline	−1.53 ± 22.12	−14.73 ± 21.20	−8.95 ± 20.44	4.785^∗^	a

Note. a = significant difference between T3 and T7; b = significant difference between T7 and T12; c = significant difference between T3 and T12; PreMax = preload maximum; PreMin = preload minimum; T = thoracic spine level. Values are expressed as mean ± standard deviation (N).

**Table 6:** Comparison of force according to the spine level on the *z*-axis in A to P TSM (*N* = 32).

	T3	T7	T12	*F*	Post hoc
Baseline	97.59 ± 67.35	33.55 ± 46.62	6.58 ± 23.13	37.460^∗^	a, b, c
Peak	833.16 ± 169.39	803.00 ± 147.73	725.90 ± 132.40	21.119^∗^	b, c
Peak-baseline	735.57 ± 148.24	769.45 ± 140.50	719.33 ± 131.73	84.381^∗^	a, b, c

Note. a = significant difference between T3 and T7; b = significant difference between T7 and T12; c = significant difference between T3 and T12; PreMax = preload maximum; PreMin = preload minimum; T = thoracic spine level. Values are expressed as mean ± standard deviation (N).

## Data Availability

The Excel data used to support the findings of this study are included within the article.
